# Tunable Hypersonic Bandgap Formation in Anisotropic Crystals of Dumbbell Nanoparticles

**DOI:** 10.1021/acsnano.3c05750

**Published:** 2023-09-27

**Authors:** Hojin Kim, Abdellatif Gueddida, Zuyuan Wang, Bahram Djafari-Rouhani, George Fytas, Eric M. Furst

**Affiliations:** † Department of Chemical & Biomolecular Engineering, University of Delaware, Newark, Delaware 19716, United States; ‡ Institut d’Electronique, de Microélectronique et de Nanotechnologie (IEMN), UMR-CNRS 8520, Département de Physique, Université de Lille, F-59655, Villeneuve d’Ascq, France; § Max Planck Institute for Polymer Research, Ackermannweg 10, 55128 Mainz, Germany; ∥ Institute of Electronic Structure and Laser, Foundation for Research and Technology-Hellas (FORTH), 71110 Heraklion, Greece

**Keywords:** Directed self-assembly, Anisotropic crystal, Phononic materials, Brillouin light scattering, Metamaterials

## Abstract

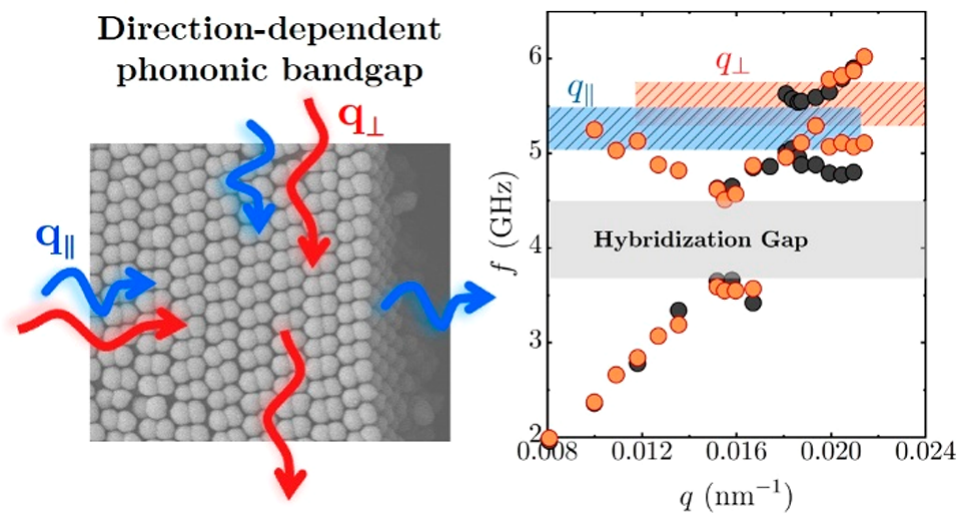

Phononic materials exhibit mechanical properties that alter the propagation of acoustic waves and are widely useful for metamaterials. To fabricate acoustic materials with phononic bandgaps, colloidal nanoparticles and their assemblies allow access to various crystallinities in the submicrometer scale. We fabricated anisotropic crystals with dumbbell-shaped nanoparticles via field-directed self-assembly. Brillouin light spectroscopy detected the formation of direction-dependent hypersonic phononic bandgaps that scale with the lattice parameters. In addition, the local resonances of the constituent nanoparticles enable metamaterial behavior by opening hybridization gaps in disordered structures. Unexpectedly, this bandgap frequency is robust to changes in the dumbbell aspect ratio. Overall, this study provides a structure–property relationship for designing anisotropic phononic materials with targeted phononic bandgaps.

Directed self-assembly is a powerful means to fabricate periodic, crystalline structures by harnessing the thermodynamically driven order–disorder transitions of nanoparticles (NPs).^1^ The building blocks are interesting due to their distinct optical and mechanical properties that give rise to particle-level and collective photonic and phononic activities in materials. These properties have been used to develop functional materials including photonic crystal pigments, phononic hypersonic filters, cloaking materials, and metamaterials.^2−6^

The performance of phononic materials toward the efficient control of acoustic wave propagation depends strongly on structure; therefore, it is vital to create nanostructures to understand and tune their phononic properties. For crystals made of spherical particles, various crystalline structures including face-centered cubic (fcc), body-centered orthorhombic, hexagonal close-packed, and body-centered tetragonal phases, can be formed depending on whether an applied external magnetic or electric field is used.^7−9^

Hypersonic (GHz) phononic bandgaps have been observed previously in colloidal crystals of spherical particles with an fcc structure. The phononic bandgaps were measured by Brillouin light spectroscopy (BLS).^10,11^ The BLS technique records the phonon dispersion ω(*q*) of the angular frequency ω versus the phonon wavenumber *q* in sufficiently transparent structures for well-defined wavenumbers. Colloidal crystals of polystyrene (PS) particles exhibit phononic bandgaps in the hypersonic frequency regime, which is induced by the periodicity in elasticities of the particle lattice and constitute an interference Bragg bandgap (BG). The BG position may be varied by changing particle size, which changes the lattice parameter of the crystal. In addition to the observations of phononic bandgaps by BLS,^10−14^ thermally simulated phonon techniques, such as time-resolved picosecond ultrasonics, laser-induced transient grating,^15^ and a recently reported frequency-domain hybrid technique^16^ have been employed. Pump–probe picosecond ultrasonics provides indirect evidence of a bandgap in silica opals through a surface localized vibration with a frequency located inside the calculated phononic bandgap.^17,18^

In addition to Bragg bandgaps, another type of phononic bandgap related to avoided-crossing effects (hybridization gaps, HG) was reported for colloid-based phononics.^19^ The HG arises from local resonances of the particles. It originates from the quadrupolar (*l* = 2) resonance of individual particles in a liquid matrix.^20^ The HG is, therefore, robust to disorder and occurs at phonon wavelengths longer than the structure spacing, leading to metamaterial behavior.^20−22^ However, inevitable interparticle contacts strongly affect the long-wavelength speed of sound and the nature of the particle vibration resonance-induced HG bandgap. It appears that the interfacial contact changes the origin of the HG from the quadrupolar (*l* = 2) to dipole (*l* = 1) particle resonance in both SiO_2_ (hard) and poly(methyl methacrylate) (soft) colloidal crystals.^14^ This type of HG was reported in polymer-tethered colloids which, in contrast to the previous resonant units, exhibit an inhomogeneous density profile.^23^

Although the realization of hypersonic phononic bandgaps in assembled submicron colloids is well-established,^10,11,14,17−19,23,24^ each phononic material is limited to a single bandgap position, requiring a different material in order to target a wider range of frequencies. In this work, we demonstrate that the use of anisotropic nanoparticles and their resulting crystals overcomes this limitation. The direction-dependent lattices of anisotropic crystals give rise to a tunable periodicity specific to the direction of the propagating phonons and thus bandgap frequency. Hence, multiple bandgaps can be achieved with a single material. The anisotropic structure is expected to exhibit different phononic activities compared to symmetric crystals of isotropic spheres.^1,25^ In addition, this anisotropic crystal should differ from ordered prolate ellipsoids where the BG is missing due to the absence of translational order, although such ellipsoidal colloidal films do form hybridization bandgaps that depend on the particle aspect ratio and orientation.^24^

We use anisotropic colloidal crystals fabricated from dumbbell-shaped NPs (Figure 1a) and study the formation of anisotropic phononic bandgaps. First, the hybridization bandgaps of the colloidal crystals are measured to elucidate the effect of complex vibrational eigenmodes of the dumbbell particles on the bandgap formation. Notably, the lowest frequency eigenmode of dumbbell-shaped NPs is the out-of-phase dipolar vibration of the two lobes^26^ which is absent in spherical NPs. Next, the anisotropic crystal of symmetric dumbbells is measured for additional Bragg bandgap formation. In this measurement, we use BLS to record the phonon dispersion relation parallel and perpendicular to the major axis of the assembled crystal to discern the direction-dependent hypersonic bandgap formation. We show that the BG can be scaled by the lattice spacing, which leads to a structure–property relation for designing phononic crystals.

**Figure 1:**
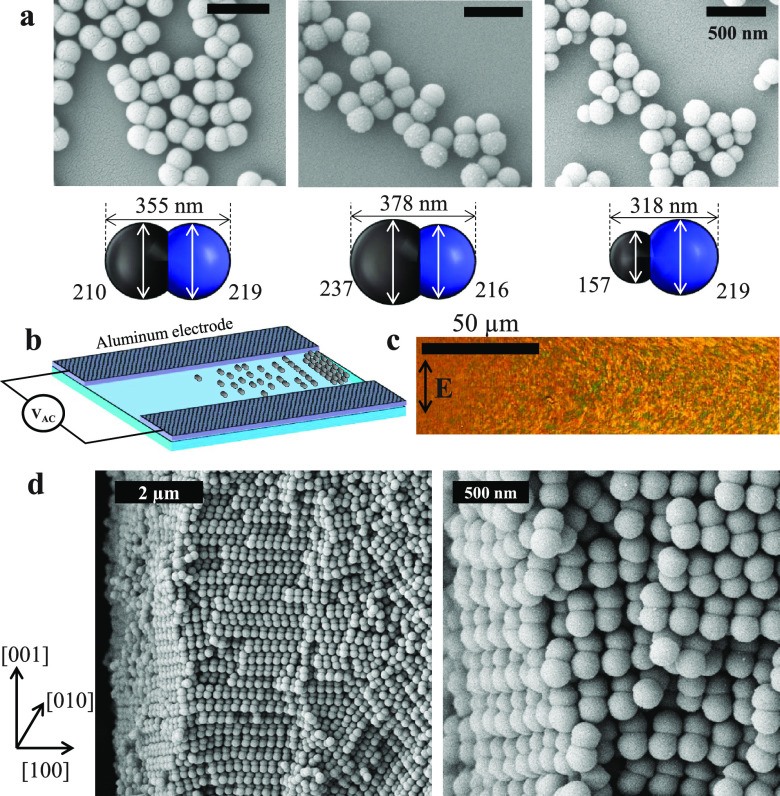
(a) Scanning electron microscopy (SEM) images of dumbbell-shaped nanoparticles. The size difference of the two lobes (black for the protruded lobe and blue for the seed lobe) increases from left to right; DB1.05, DB1.10, and DB1.40. The estimated dimension (lobe diameters *d*_1_, *d*_2_, and length *L*) of each particle is shown below the SEM images. (b) A schematic illustration of a coplanar electrode cell for self-assembling dumbbells. The medium of the dumbbell suspension evaporates from both ends, and the assembled crystallites are deposited at one end, forming a millimeter-scale colloidal crystal. (c) As crystallites are deposited, strong birefringence appears. The applied field direction in this image is from bottom to top. (d) SEM images of a crystal of self-assembled dumbbell particles (DB1.05) at low (left) and high (right) magnification. The crystal is fabricated in the presence of AC electric field with the direction from bottom to top.

## Results and Discussion

Three dumbbell particles were synthesized by a two-step seeded emulsion polymerization, controlling their symmetry and anisotropy by varying the amount of swelling monomer (synthesis details are provided in the Methods).^27^ We vary the aspect ratio of the dumbbells (Figure 1a); the diameter ratio *d*_1_/*d*_2_ of the larger (*d*_1_) and the smaller (*d*_2_) lobes is 1.05, 1.10, and 1.40 (from left to right), respectively. The dumbbell length *L,* also obtained from scanning electron microscopy images, increases by 19% from DB1.40 to DB1.10. The dumbbell NPs were assembled by a field-directed self-assembly technique (Figure 1b) to avoid the randomly packed structure anisotropic particles exhibit at high volume fractions.^28^ The particles align and form small crystallites in suspension under an AC electric field. During the field-directed assembly, the suspending medium (water) simultaneously evaporates, leading to the deposition of the crystallites into millimeter-scale crystals. The formation of highly ordered crystals was accompanied by strong optical birefringence (Figure 1c). It shows a density gradient of the birefringence (i.e., crystallites) due to the convective deposition from left to right, perpendicular to the direction of the applied electric field (vertical arrow, Figure 1c). In this way, symmetric dumbbell NPs (DB1.05) formed a 3D anisotropic crystal with both translational and orientational order. Scanning electron microscopy images confirmed the expected monoclinic crystalline structure (Figure 1d).^28^ Disordered films are deposited using the same convective assembly method in the absence of an applied field (Figure S1).

### Hybridization Bandgap Formation in Disordered Dumbbells

In assemblies of spherical colloids, hybridization bandgaps are robust to structural disorder.^14,19^ Hence, we examine disordered colloidal films of the three dumbbell NPs infiltrated with the poly(dimethylsiloxane) (PDMS) fluid. The PDMS has a refractive index (*n* = 1.45) that is close to that of the PS dumbbells (*n* = 1.59). The PDMS infiltration reduces multiple light scattering and creates a condition in which the *q*-dependent BLS measurements can record the dispersion relation. In addition, the infiltrated close-packed dumbbell NPs have a large elastic impedance contrast due to the low longitudinal sound velocity (1050 m s^–1^) in PDMS compared to PS (2380 m s^–1^).

**Figure 2:**
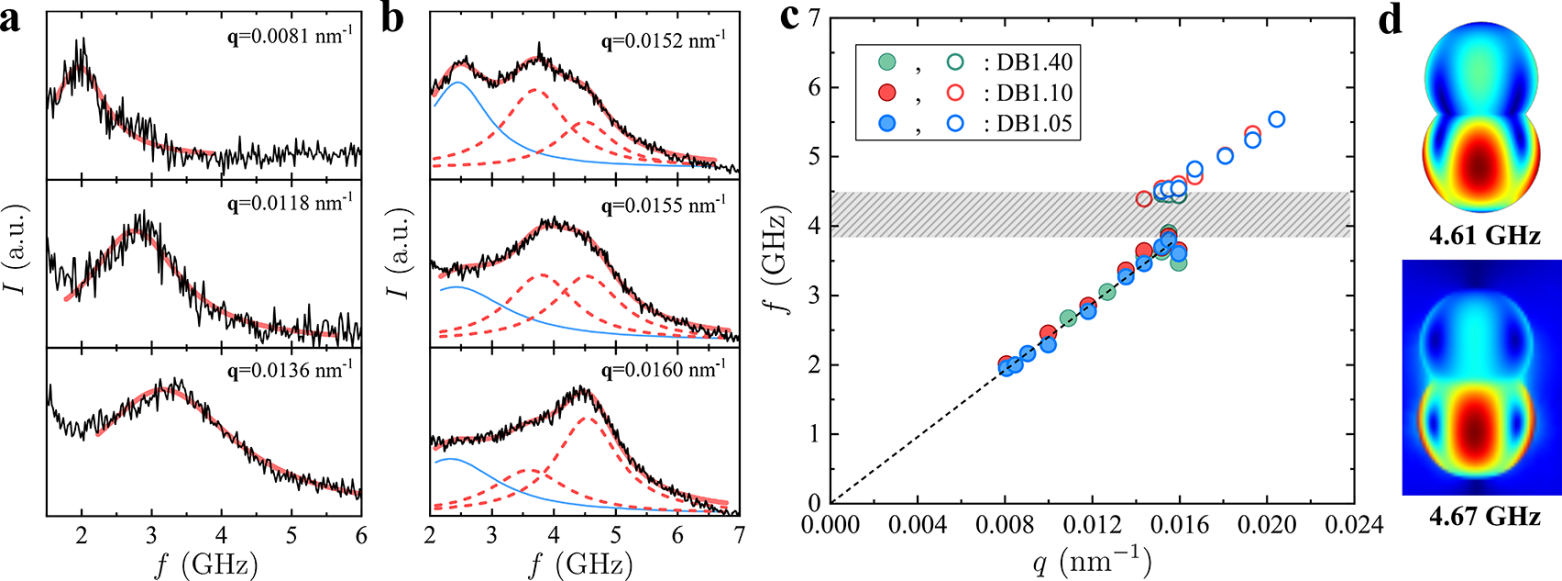
BLS spectra (a) below the bandgap of randomly packed dumbbells, DB1.05, and (b) near the hybridization bandgap at three different scattering wavevectors q. The red solid line indicates the representation of the BLS spectra by (a) a single and (b) two Lorentzian line shapes (dashed lines). (c) The phonon dispersion relation of DB1.05 (blue), DB1.10 (red), and DB1.40 (green) with a hybridization bandgap (hatched area). The dashed line indicates the acoustic branch at low scattering vectors. (d) Finite element calculations of quadrupolar eigenmodes in air (top) and in PDMS (bottom) with a frequency of 4.61 and 4.67 GHz, respectively.^26^

In the BLS transmission geometry, the scattering vector **q** is parallel to the film with *n*-independent magnitude *q* = (4π/λ) sin (θ/2), where λ = 532 nm is the vacuum wavelength of the laser and the scattering angle θ = 2α, with α being the incident angle of the beam.^11^Figure 2a presents BLS spectra of the randomly packed DB1.05 film in the low-*q* regime (see Figure S2 for BLS spectra of DB1.10 and DB1.40), which has an acoustic phonon band (dotted line in panel Figure 2c). As *q* increases, the broadened peak indicates the contribution of an additional band. With a further increase in *q*, the peak splits, and the BLS spectra are represented by two Lorentzian lines (red dashed lines in Figure 2b). Above this, the intensity of the low frequency acoustic branch decreases, and the upper frequency peak intensifies. The spectral splitting above approximately *q* = 0.014 nm^–1^ indicates the emergence of a hybridization phononic bandgap centered at *f*_HG_ ∼ 4.1 GHz and *q*_HG_ ≈ 0.015 nm^–1^ in Figure 2c. Notably, the position of the HG remains the same for all three disordered DB films with different particle geometries.

The phonon dispersion relation of DB1.05 in Figure 2c has an acoustic branch with the effective sound velocity *c*_eff_ = 2π*f*/*q* = 1500 ± 30 m s^–1^ in the PDMS-infiltrated disordered films. The value of *c*_eff_ is between the longitudinal sound velocity of PS and PDMS and compares well with the estimated value (1520 m s^–1^) from Wood’s law, assuming a PS filling fraction equal to 0.67. This close agreement implies weak particle interactions in contrast to the spherical counterparts in fcc colloidal crystals that have a higher *c*_eff_ = 1670 ± 30 m s^–1^ in the same PDMS matrix.^19^

The effective sound velocity is an important feature of the phonon dispersion measured by BLS. We performed finite element simulations to obtain the theoretical value and study its dependence on the interactions between the dumbbell nanoparticles. For the calculation, a monoclinic structure of dumbbells (Figure 1d) is used, and the lattice constants *a*_1_ and *a*_2_ (*a*_1_ = *a*_2_) are varied at fixed *a*_3_ = 392 nm (particle major axis, see Figure S3 for detail lattice geometry). At  nm (>*d*_1_ = 219 nm), dumbbells are not in direct contact. The longitudinal sound velocity for noninteracting dumbbell nanoparticles embedded in PDMS is computed to be *c*_eff_ ≈ 1307 m s^–1^ (approximately 13% lower than the experimental value) and, unexpectedly, the same for both [110] (perpendicular to the applied field) and [001] (parallel to the applied field) directions.

In this configuration of a phononic crystal of solid scatterers immersed in a liquid, there is only one acoustic branch. It originates from the propagation in the liquid; therefore, the corresponding velocity is close to the speed of sound in the liquid. In contrast, if NPs slightly overlap, a qualitative change arises to the dispersion curves, and now four acoustic branches start from zero. The highest, which is essentially the longitudinal mode of the phononic crystal, is most likely the one observed in BLS and has a significantly higher velocity. For instance, a decrease in *a*_1_ and *a*_2_ to  nm (<*d*_1_) leads to the contact at the larger lobe, and the effective sound velocity along [110] increases to 1405 m s^–1^; the change in the velocity along [001] remains modest as there is no overlap along this direction. A further decrease to  nm (<*d*_2_) results in complete overlap between dumbbells at both the small and big lobes, in which PDMS pockets form inside the PS network, and the effective sound velocity increases to 1525 m s^–1^. Therefore, mechanical interactions (i.e., overlapping nanoparticles) are necessary to achieve a theoretical effective sound velocity comparable to that of the experimental one. We have found similar theoretical trends in the case of fcc crystals of PS spheres, where the jump from the liquid-like acoustic branch to the longitudinal branch results in an abrupt increase in the effective sound velocity as particles begin to overlap even less than 1%.^14^

Returning to the hybridization bandgap, the HG width, Δ*f*/*f*_HG_ ∼ 12% resolves due to the strong resonance of the dumbbell particles in the PDMS matrix. HG in disordered spherical colloid films is caused by an anticrossing of two bands with the same symmetry and occurs at *q*_HG_*d* ∼ 3.2 and *f*_HG_*d*/*c*_eff_ ∼ 0.5 with *d* being the NP diameter.^14,19^ For the quasi-spherical DB1.05 with seed lobe diameter *d*_1_ = 219 nm, *q*_HG_*d* ∼ 3.3 and *f*_HG_*d*/*c*_eff_ ∼ 0.6 resemble the HG position in films of close-packed spherical colloids. The values for the HG, *q*_HG_ and *f*_HG_, in asymmetric dumbbells (DB1.10 and DB1.40) are in good agreement with the symmetric dumbbell DB1.05, implying that the HG is controlled by the size of the seed lobe in the three DB films. Comparable quadrupolar modes occur in air, too.^26^

The calculated quadrupolar eigenmodes of DB1.05 in air and in PDMS medium are shown in Figure 2d (see Table S1 for the quadrupolar modes of DB1.10 and DB1.40). The frequency of the quadrupolar eigenmodes *f* ∼ 4.6 GHz is somewhat higher than the experimental value *f*_HG_ ∼ 4.1 GHz. The eigenfrequencies also exhibit a weak dependence on the infiltrating medium (4.61 GHz in air and 4.67 GHz in PDMS) and aspect ratio, in contrast to the corresponding mode *f*(*l* = 2) (the angular dependence of the displacement *l*) of spherical PS NPs^19^ which exhibit a strong dependence on the medium. Next, considering the dipolar vibrational modes of the dumbbells, the low-frequency modes strongly depend on the aspect ratio and, moreover, are red-shifted in PDMS; the frequency of the dipolar mode increases from 3.0 GHz for DB1.05 to 3.5 GHz for DB1.40.^26^

The HG frequency for all three dumbbells (Figure 2c) is well above the dipolar mode frequency, and an anticrossing between the dipolar mode and the effective medium phonon is apparently inactive. Nonetheless, the proximity of *f*_HG_ and the broad quadrupolar eigenmode frequencies may reflect an anticrossing mechanism to these NP resonances.^26^ Dumbbells were synthesized from the same seed sphere (blue in Figure 1b), but the protruding lobe size varies (black in Figure 1b). As a result, dumbbells with three different asymmetries share comparable quadrupolar modes responsible for the vibration of the seed lobe (*d*_1_ ∼ 219 nm), leading to identical HG positions. In this case, the HG in Figure 2c occurs at the reduced frequency *f*_HG_*d*/*c*_eff_ ∼ 0.6. Note that the origin of HG in close-packed PS spherical colloids in PDMS changes from a quadrupolar (*l* = 2) to dipole (*l* = 1) particle resonance for NPs with strong interfacial contact;^14^ for the former, *f*(*l* = 2)*d*/*c*_eff_ ∼ 0.31. For both colloidal particles, HG is a property of metamaterials, and hence, it occurs at wavelengths 2π/*q*_HG_ (∼420 nm) larger than the structure periodicity.

### Anisotropic Bragg Bandgap

The presence of a hypersonic Bragg bandgap has been reported in crystals of spherical colloids^11,19^ and other periodic structures;^29^ therefore, a crystal of symmetric dumbbell NPs is expected to similarly exhibit BG. We examined the phonon dispersion along orthogonal directions of the anisotropic crystalline structure (see the SEM image in Figure 1d). Due to the vector nature of **q**, BLS records the phonon dispersion with the phonon scattering vector **q** parallel (*q*_∥_) and perpendicular (*q*_⊥_) to the AC electric field that directs the crystal assembly. As the dumbbell NPs align with the applied field direction, *q*_∥_ refers to the orientation of the long dumbbell long axis. The assembled crystal grows in the *z*-direction; the height depends on the assembly channel height governed by the approximately 20 μm thick spacer. Presumably, a higher stacking of particles in the *z*-direction can be fabricated. However, the dielectrophoretic assembly works best when the height is a fraction of the electrode separation. Hence, the phonon dispersion in the *z*-direction (normal to the *q*_∥_-*q*_⊥_ plane) has not yet been explored yet.

**Figure 3:**
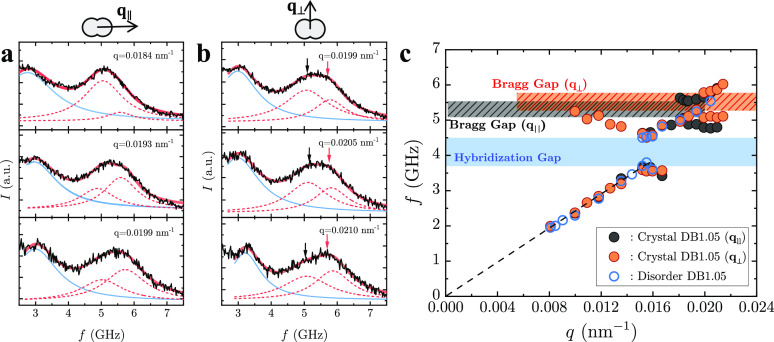
BLS spectra at q parallel (*q*_∥_) (a) and perpendicular (*q*_⊥_) (b) to the particle long axis near the Bragg bandgap of the self-assembled DB1.05 dumbbell crystal. BLS spectra (black solid line) are represented (red solid line) by double Lorentzian lines (red dashed line). The low frequency peak (blue solid line) is for the acoustic phonon of the PDMS atop the crystal. (c) The phonon dispersion relation of a self-assembled DB1.05 colloidal crystal recorded along (*q*_∥_, black) and normal (*q*_⊥_, orange) to the dumbbell long axis. Blue filled area indicates the hybridization bandgap along both directions. Orange and black areas refer to the Bragg bandgaps for *q*_⊥_ and *q*_∥_, respectively. A dotted line indicates the linear q dependence of the frequency for the acoustic branch.

Figure 3a and 3b presents BLS spectra of the crystalline assembly of DB1.05 dumbbell in the high *q*-range (*q* > *q*_HG_) recorded for *q*_∥_ and *q*_⊥_, respectively. For *q* < *q*_HG_ in the linear acoustic regime and *q* ∼ *q*_HG_ in the HG region, the BLS spectra are indistinguishable from those in the disordered structure (Figure S2 and Figure S4). Notably, the acoustic branch of the experimental phonon dispersions exhibits a weak dependence on both the crystalline order and the phonon propagation direction, leading to a well-resolved effective sound velocity. In contrast, the effective sound velocities from the calculated phonon dispersions clearly show a direction dependence, especially for cases with overlapping dumbbell nanoparticles along [110]. However, an inclusion of overlap along [001] with a suitable amount of overlap in the orthogonal direction might eliminate this direction dependent sound velocity.

At higher scattering vectors near the Brillouin zone (*q*_BZ_ ∼ 0.019 nm^–1^) along either direction (Figure 3a for *q*_∥_ and Figure 3b for *q*_⊥_), the spectra begin to broaden and become asymmetric compared to the disordered state (Figure S5). In addition, at the same *q* (= 0.0199 nm^–1^), the crystalline and disordered spectra for DB1.05 do not overlap. However, in contrast to the spectra at lower *q* ∼ *q*_HG_, there is no apparent line splitting in the BLS spectra of the DB1.05 crystal at higher *q* ∼ *q*_BZ_ mainly due to phonon multiple scattering plus the inherent broadening that scales with *q*^2^.^30^ Hence, we represent the spectra in the crystalline DB1.05 with a double Lorentzian line shape, in addition to the PDMS peak (Figure 3a and 3b). In this representation, the skewness of the peak shifts from left-sided to right-sided as *q* increases (relative contribution of black and red arrows in Figure 3b), which is a strong indication of bandgap formation. This analysis reveals a second bandgap at higher *q* (shorter lengths) than *q*_BZ_. Note that a single Lorentzian representation of the crystalline DB1.05 along both directions, as for the disordered sample for *q* > *q*_HG_, smears out this second bandgap. Therefore, we focus on the result of two-peak analysis (see Figure S6 for the single-peak analysis). In the peak fitting procedure, we added the peak of the PDMS infiltrating medium in the low frequency regime (blue line) to reduce the effect of PDMS background intensity and enhance the fit quality.

The band diagram for crystalline DB1.05 along the *q*_∥_ (black) and *q*_⊥_ (orange) directions is shown in Figure 3c. There are two discrete Bragg bandgaps corresponding to the two wavevector directions (*q*_∥_ and *q*_⊥_) near the Brillouin zone; the center of the BG, *f*_BG_, for *q*_∥_ and *q*_⊥_ is *f*_BG_ = 5.2 and 5.5 GHz, respectively. The bandgap width Δ*f*_BG_ = 0.5 GHz is similar in both cases, i.e., about 10% of the normalized bandwidth. Notably, the Bragg gaps for two different wavevector directions overlap at around 5.5 GHz, indicating a possible full bandgap in the *q*_∥_-*q*_⊥_ plane. The origin of the anisotropic BGs is associated with the two different periodicities probed along the orthogonal directions. We can rationalize the phenomenon semiquantitatively by using the crystal lattice constants. Even if the specific structure of assembled dumbbells has not been demonstrated, lattice structures can be identified by using crystal structures formed by submicrometer-scale dumbbells including tetragonal, base-centered monoclinic, and body-centered tetragonal phases.^31^ Based on the SEM image in Figure 1d, dumbbell particles are tilted from the applied AC electric field direction, which indicates a base-centered monoclinic structure. Given the aspect ratio *L*/*d* = 1.7 of DB1.05, the ratio of the particle length (*L*) to the diameter of a bigger lobe (*d*_1_), the tilted angle of particles (β) is approximately β = 26^◦^. The lattice constant (*a*) of base-centered monoclinic structure varies depending on its direction; *a* parallel (*a*_∥_) and perpendicular (*a*_⊥_) to the AC electric field direction. For the estimation of the lattice constant, the tilted geometry in the crystal is considered. According to the crystal lattice structure shown in Figure S3, *a*_∥_ = *L* cos β (= 320 nm) and  (= 300 nm) with *d* being the average diameter of two lobes *d*_1_ and *d*_2_.

**Figure 4:**
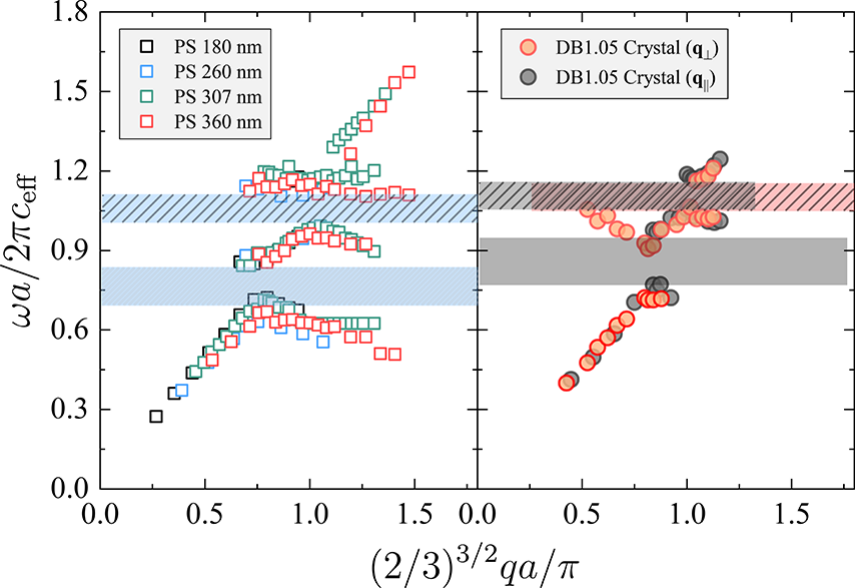
Phonon dispersion relation is presented with reduced axes, *ωa*/2*πc*_eff_ versus (2/3)^3/2^*qa*/π, for colloidal crystals consisting of PS (left) spheres and (right) dumbbells. The reduced plot of PS spheres with diameter *d* = 180 nm (black), 260 nm (blue), 307 nm (green), and 360 nm (red) is taken from earlier experiments.^19,32^ The phonon dispersion relations of dumbbell crystal at *q*_∥_ (black circle) and *q*_⊥_ (orange circle) are reduced with a lattice constant of *a*_∥_ = *L* cos β and , respectively. Filled and hatched areas indicate the normalized HG and BG positions, respectively. Blue, black, and red dashed areas indicate the collapsed Bragg bandgap of fcc sphere and dumbbell crystal at *q*_∥_ and *q*_⊥_, respectively. Blue and black filled areas refer to the hybridization bandgap of the fcc sphere and dumbbell crystal at *q*_∥_ and *q*_⊥_ (average range of HG), respectively.

In the case of BG and similar packing ratio, the dispersion relation of fcc crystals of spherical PS colloids overlaps when plotted in the reduced form,^11^*ωa*/2*πc*_eff_ versus *q*/*q*_BZ_, where, along ΓΜ direction, the relation *q*_BZ_ = *q*_ΓM_ = π/[(2/3)^3/2^α] holds, as shown in the left panel of Figure 4. Spherical colloids have a single dimension (i.e., particle diameter *d*), and lattice constant ; therefore, the phonon dispersion simply scales with the particle size. A BG occurs at *q*/*q*_BZ_ ≈ 1 and ω_BZ_α/2π*c*_eff_ ≈ 1 and HG at longer phonon wavelength, *q*/*q*_BZ_ ≈ 0.75 at ω_HG_α/2π*c*_eff_ ≈ 0.75. Note that an assignment of the HG to the sphere quadrupolar (*l* = 2) would appear at ω(*l* = 2)*d*/*c*_eff_ ∼ 0.31, clearly at a lower reduced frequency. By contrast, dumbbell particles have multiple geometric parameters, and their lattice constants depend strongly on the direction of propagating phonon.

Our consideration of the anisotropic lattice constants along each direction successfully collapses the BGs from both parallel (*q*_∥_, black area) and perpendicular (*q*_⊥_, red area) directions. BG and HG (hatched areas) overlap at about 10% higher values compared with the spherical particles observed previously.^11,19,32^ The HG due to the dumbbell quadrupolar mode (Figure 2d) would appear at  exceeding the experimental reduced frequency (≈0.85). On a premise of superposition, the simplest account for this small difference could be a renormalization of *d* that directly impacts HG and BG, at least in the perpendicular direction. However, a detailed discussion of the subtle disparity in Figure 4 is limited by the geometric and structural complexities of the crystal. In contrast to the experimental phonon dispersions, the theoretical phononic band structure includes more branches and exhibits no identifiable band gap. The experimentally observed band gaps could be attributed to the inactivity of some of the phonon modes, as prescribed by the selection rules of phonon scattering and the photoelastic constants of the sample. Then, the few branches with significant BLS activity may present an avoided-crossing, analogous to the experimental bandgap. Nonetheless, the normalization of phonon dispersion in the reduced axis clearly overlaps the two discrete bandgaps, regardless of different lattice structures: fcc versus monoclinic structure. This structure-independent normalization suggests a unified structure–property relation for colloidal phononic crystals.

## Conclusions

In this work, we demonstrated that the anisotropic colloidal crystal of symmetric dumbbell nanoparticles exhibits anisotropic phononic bandgaps of hypersonic waves. By measuring the dispersion relation at two orthogonal directions, we found that the position of the Bragg phononic bandgap depends on the propagating direction of the waves relative to the crystal lattice. A Bragg bandgap results from the periodic boundary conditions; its location can be normalized with the lattice parameters for each direction (parallel and perpendicular to the major axis of the dumbbell particles). Hence, the reduced phonon dispersion relation provides a structure–property relation that can be used to further design and tailor positions of the phononic bandgap of self-assembled colloidal crystals with complex structure.

In contrast to the Bragg bandgap, the position of the hybridization gap is robust, as expected, to disorder and to the particle symmetry. A comparison of the hybridization gaps of three different dumbbell-shaped particles revealed that the quadrupolar mode of the seed lobe of the dumbbell is the underlying mechanism for its existence. This assignment is supported by the value of effective medium velocity, which supports solid–liquid topology conforming to Wood’s mixture law. However, further predictions of the Bragg bandgap position were hampered by the sensitivity of the band structure to the particle geometry, packing, and configuration.

Overall, our findings present a structure-phononic property relation with anisotropic phononic bandgaps from monoclinic structures of colloidal crystals. These results point to opportunities to advance theory and modeling for the design of functional colloidal phononic materials. Other difficulties that are encountered when engineering defect-free colloidal crystals, especially the presence of twinning defects in fcc packings of spheres, can be avoided by using anisotropic building blocks and their crystalline structures, such as the simple monoclinic structure of dumbbell nanoparticles.

## Methods

### Particle Synthesis

Dumbbell-shaped particles were synthesized by two-step emulsion polymerization.^27,33,34^ Briefly, the seed PS nanoparticles with *d* = 140 nm were prepared by surfactant-free emulsion polymerization.^35^ For the core/shell structure, 15 mL of the seed particles (10 wt % in water) was diluted with 12 mL of water (Millipore Direct-Q 5 ultra purification system, resistivity >18.2 MΩcm), and the suspension was mixed with a mixture of 1.6 mL of styrene (St, Sigma-Aldrich), 0.2 mL of 3-(trimethoxysilyl) propyl acrylate (TMSPA, Sigma-Aldrich), and 0.01 g of azobis(isobutyronitrile) initiator (AIBN, Sigma-Aldrich). In a glass vial, the mixture was vortexed for 10 min at room temperature. The vial was loaded to a mechanical stirrer and immersed in a water bath with temperature *T* = 70 °C. Initiation of polymerization was performed for 8 h with a stirring rate of 300 rpm. Synthesized PS/poly(St-*co*-TMSPA) core/shell particles were again polymerized to form dumbbell-shape. Then 11.5 mL of water was added to 8 mL of PS/poly(St-*co*-TMSPA) particle suspension and 1 mL of F 108 (BASF) surfactant aqueous solution (1 wt %). Next, 0.007 g of sodium 4-vibylbenzenesulfonate (Sigma-Aldrich) was added to the solution. The core/shell particles were swollen by mixing the suspension with a solution of St monomer (0.95 mL) and AIBN initiator (0.005 g). The aspect ratio of dumbbell particles was controlled by the amount of St monomer. After stirring for 10 min at room temperature, monomer was polymerized for 8 h at 70 °C. The particles were washed by centrifugation at least three times.

### Directed Self-Assembly of Dumbbell Nanoparticles

To assemble the dumbbell nanoparticles into colloidal crystals, an AC electric field was applied over the particle suspension. Aluminum coplanar electrodes with a 1 mm gap were photolithographically fabricated with a photomask (Fineline Imaging) by depositing 100 nm aluminum vapor on 25 × 25 mm glass coverslips (Fisher NC1811325).^36^ The mask was lifted off in acetone, and the electrodes were washed with 2-propanol (Fisher). Before the self-assembly process, electrodes and glass slides were washed in a NoChromix (Sigma-Aldrich) and sulfuric acid (Fisher) solution overnight and rinsed with water. After drying with nitrogen gas, they were plasma cleaned. On a glass slide (Fisher, 15 × 75 mm), the aluminum electrode was attached with a 20 μm spacer made of a mixture of UV curable glue (Norland Optical Adhesive 81) and 20 μm diameter silica particles (Sigma-Aldrich).^37^ A suspension of the dumbbell particles (volume fraction, = 0.15) was pipetted into the gap between electrodes by capillary action, and an AC electric field with a field strength of *E* = 154 V cm^–1^ and a frequency of *f* = 150 kHz was applied over the suspension. Dumbbell particle crystallites were deposited by evaporating the medium (water) through the open ends in the presence of electric fields in the manner demonstrated for anisotropic titania nanoparticles by Mittal and Furst.^38^ Randomly packed dumbbell films were prepared by evaporating a dumbbell suspension under no electric field.

### Characterization

The self-assembly process was observed with an optical microscope (Microscope Axio Observer Ai Zeiss AXIO), and images were taken with a digital camera (Canon-EOS Digital Rebel T2i). The fabricated crystal and randomly packed film of dumbbells were imaged with a scanning electron microscope (JEOL JSM-7400F) with 3 kV of accelerating voltage.

### Brillouin Light Scattering (BLS)

The eigenmodes of particles and the phonon dispersion relation were measured with BLS. BLS is inelastic light scattering, which nondestructively probes the thermal density fluctuations of materials. The scattering wave vector (*q*) is defined as ± *q* = *k*_s_ – *k*_i_, where *k*_s_ and *k*_i_ refer to the scattered and incident light vector. The measured BLS spectra of transparent colloidal film show a single doublet associated with Doppler shift, and the magnitude of frequency at q is defined as *f*_l, t_ = ± *c*_l, t_*q*/2π, where *c* is the sound velocity (subscript l and t refer to longitudinal and transverse wave, respectively). The wave vector *q* in the transmission geometry has the magnitude  with the wavelength of incident light, λ = 532 nm, and scattering angle θ. It is notable that the magnitude of *q* is independent of the refractive index of the medium. For the transparent colloidal films for *q*-dependent measurements, PDMS fluid (MW = 770 Da) was infiltrated in the opaque dried colloidal film, and the excess amount of PDMS is removed by blowing nitrogen gas overnight.

## Supporting Information Available

Supporting Figures: Scanning electron microscopy images of disordered colloidal films; BLS spectra of disordered colloidal films; lattice analysis of the crystal structure; BLS spectra of the colloidal crystal near the hybridization bandgap; peak width and skewness analysis of the colloidal crystal; one-peak analysis of the colloidal crystal near the Bragg bandgap. Supporting Table: finite element analysis of the eigenfrequencies of dumbbell particles (PDF)

## References

[ref1] Grzelczak M.; Vermant J.; Furst E. M.; Liz-Marzán L. M. Directed Self-Assembly of Nanoparticles. ACS Nano 2010, 4 (7), 3591–3605. 10.1021/nn100869j. 20568710

[ref2] Maldovan M. Phonon Wave Interference and Thermal Bandgap Materials. Nat. Mater. 2015, 14 (7), 667–674. 10.1038/nmat4308. 26099716

[ref3] Maldovan M. Sound and Heat Revolutions in Phononics. Nature 2013, 503 (7475), 209–217. 10.1038/nature12608. 24226887

[ref4] Cheng W.; Gorishnyy T.; Krikorian V.; Fytas G.; Thomas E. L. In-Plane Elastic Excitations in 1D Polymeric Photonic Structures. Macromolecules 2006, 39 (26), 9614–9620. 10.1021/ma062109i.

[ref5] Gorishnyy T.; Ullal C. K.; Maldovan M.; Fytas G.; Thomas E. L. Hypersonic Phononic Crystals. Phys. Rev. Lett. 2005, 94 (11), 1–4. 10.1103/PhysRevLett.94.115501. 15903869

[ref6] Aguirre C. I.; Reguera E.; Stein A. Colloidal Photonic Crystal Pigments with Low Angle Dependence. ACS Appl. Mater. Interfaces 2010, 2 (11), 3257–3262. 10.1021/am100704f. 20964439

[ref7] Hynninen A. P.; Dijkstra M. Phase Diagram of Dipolar Hard and Soft Spheres: Manipulation of Colloidal Crystal Structures by an External Field. Phys. Rev. Lett. 2005, 94 (13), 8–11. 10.1103/PhysRevLett.94.138303. 15904046

[ref8] Yethiraj A.; van Blaaderen A. A Colloidal Model System with an Interaction Tunable from Hard Sphere to Soft and Dipolar. Nature 2003, 421, 513–517. 10.1038/nature01328. 12556887

[ref9] Swan J. W.; Bauer J. L.; Liu Y.; Furst E. M. Directed Colloidal Self-Assembly in Toggled Magnetic Fields. Soft Matter 2014, 10 (8), 1102–1109. 10.1039/C3SM52663A. 24795962

[ref10] Zhu G.; Swinteck N. Z.; Wu S.; Zhang J. S.; Pan H.; Bass J. D.; Deymier P. A.; Banerjee D.; Yano K. Direct Observation of the Phonon Dispersion of a Three-Dimensional Solid/Solid Hypersonic Colloidal Crystal. Phys. Rev. B 2013, 88, 144307 10.1103/PhysRevB.88.144307.

[ref11] Cheng W.; Wang J.; Jonas U.; Fytas G.; Stefanou N. Observation and Tuning of Hypersonic Bandgaps in Colloidal Crystals. Nat. Mater. 2006, 5 (10), 830–836. 10.1038/nmat1727. 16951677

[ref12] Gorishnyy T.; Jang J.; Koh C.; Thomas E. L. Direct Observation of a Hypersonic Band Gap in Two-Dimensional Single Crystalline Phononic Structures. Appl. Phys. Lett. 2007, 91, 121915 10.1063/1.2786605.

[ref13] Gorishnyy T.; Maldovan M.; Ullal C.; Thomas E. Sound Ideas. Phys. World 2005, 18 (12), 24–29. 10.1088/2058-7058/18/12/30. 15903869

[ref14] Cang Y.; Sainidou R.; Rembert P.; Magnabosco G.; Still T.; Vogel N.; Graczykowski B.; Fytas G. Origin of the Acoustic Bandgaps in Hypersonic Colloidal Phononics: The Role of the Elastic Impedance. J. Phys. Chem. B 2022, 126 (34), 6575–6584. 10.1021/acs.jpcb.2c03923. 35997523 PMC9442645

[ref15] Hiraiwa M.; Abi Ghanem M.; Wallen S. P.; Khanolkar A.; Maznev A. A.; Boechler N. Complex Contact-Based Dynamics of Microsphere Monolayers Revealed by Resonant Attenuation of Surface Acoustic Waves. Phys. Rev. Lett. 2016, 116 (19), 198001 10.1103/PhysRevLett.116.198001. 27232047

[ref16] Rolle K.; Yaremkevich D.; Scherbakov A. V.; Bayer M.; Fytas G. Lifting Restrictions on Coherence Loss When Characterizing Non-Transparent Hypersonic Phononic Crystals. Sci. Rep. 2021, 11 (1), 17174 10.1038/s41598-021-96663-3. 34433886 PMC8387379

[ref17] Akimov A. V.; Tanaka Y.; Pevtsov A. B.; Kaplan S. F.; Golubev V. G.; Tamura S.; Yakovlev D. R.; Bayer M. Hypersonic Modulation of Light in Three-Dimensional Photonic and Phononic Band-Gap Materials. Phys. Rev. Lett. 2008, 101, 033902 10.1103/PhysRevLett.101.033902. 18764257

[ref18] Salasyuk A. S.; Scherbakov A. V.; Yakovlev D. R.; Akimov A. V.; Kaplyanskii A. A.; Kaplan S. F.; Grudinkin S. A.; Nashchekin A. V.; Pevtsov A. B.; Golubev V. G.; Berstermann T.; Brüggemann C.; Bombeck M.; Bayer M. Filtering of Elastic Waves by Opal-Based Hypersonic Crystal. Nano Lett. 2010, 10 (4), 1319–1323. 10.1021/nl904126m. 20232893

[ref19] Still T.; Cheng W.; Retsch M.; Sainidou R.; Wang J.; Jonas U.; Stefanou N.; Fytas G. Simultaneous Occurrence of Structure-Directed and Particle-Resonance- Induced Phononic Gaps in Colloidal Films. Phys. Rev. Lett. 2008, 100, 194301 10.1103/PhysRevLett.100.194301. 18518452

[ref20] Psarobas I. E.; Modinos A.; Sainidou R.; Stefanou N. Acoustic Properties of Colloidal Crystals. Phys. Rev. B 2002, 65 (6), 064307 10.1103/PhysRevB.65.064307.

[ref21] Lemoult F.; Kaina N.; Fink M.; Lerosey G. Wave Propagation Control at the Deep Subwavelength Scale in Metamaterials. Nat. Phys. 2013, 9 (1), 55–60. 10.1038/nphys2480.

[ref22] Brunet T.; Zimny K.; Mascaro B.; Sandre O.; Poncelet O.; Aristégui C.; Mondain-Monval O. Tuning Mie Scattering Resonances in Soft Materials with Magnetic Fields. Phys. Rev. Lett. 2013, 111 (26), 2–6. 10.1103/PhysRevLett.111.264301. 24483797

[ref23] Alonso-Redondo E.; Schmitt M.; Urbach Z.; Hui C. M.; Sainidou R.; Rembert P.; Matyjaszewski K.; Bockstaller M. R.; Fytas G. A New Class of Tunable Hypersonic Phononic Crystals Based on Polymer-Tethered Colloids. Nat. Commun. 2015, 6, 8309 10.1038/ncomms9309. 26390851 PMC4595630

[ref24] Beltramo P. J.; Schneider D.; Fytas G.; Furst E. M. Anisotropic Hypersonic Phonon Propagation in Films of Aligned Ellipsoids. Phys. Rev. Lett. 2014, 113 (20), 1–5. 10.1103/PhysRevLett.113.205503. 25432048

[ref25] Glotzer S. C.; Solomon M. J. Anisotropy of Building Blocks and Their Assembly into Complex Structures. Nat. Mater. 2007, 6 (7), 557–562. 10.1038/nmat1949. 17667968

[ref26] Wang Z.; Kim H.; Secchi M.; Montagna M.; Furst E. M.; Djafari-Rouhani B.; Fytas G. Quantization of Acoustic Modes in Dumbbell Nanoparticles. Phys. Rev. Lett. 2022, 128 (4), 48003 10.1103/PhysRevLett.128.048003. 35148122

[ref27] Park J.-G.; Forster J. D.; Dufresne E. R. High-Yield Synthesis of Monodisperse Dumbbell-Shaped Polymer Nanoparticles. J. Am. Chem. Soc. 2010, 132, 5960–5961. 10.1021/ja101760q. 20373805

[ref28] Forster J. D.; Park J.-G.; Mittal M.; Noh H.; Schreck C. F.; O’Hern C. S.; Cao H.; Furst E. M.; Dufresne E. R. Assembly of Optical Scale Dumbbells Into Dense Photonic Crystals. ACS Nano 2011, 5 (8), 6695–6700. 10.1021/nn202227f. 21740047

[ref29] Schneider D.; Liaqat F.; El Boudouti E. H.; El Abouti O.; Tremel W.; Butt H.-J.; Djafari-Rouhani B.; Fytas G. Defect-Controlled Hypersound Propagation in Hybrid Superlattices. Phys. Rev. Lett. 2013, 111 (16), 164301 10.1103/PhysRevLett.111.164301. 24182268

[ref30] Berne B. J.; Pecora R. Dynamic Light Scattering: With Applications to Chemistry, Biology, and Physics; Courier Corporation, 2000.

[ref31] Demirörs A. F.; Johnson P. M.; van Kats C. M.; van Blaaderen A.; Imhof A. Directed Self-Assembly of Colloidal Dumbbells with an Electric Field. Langmuir 2010, 26 (18), 14466–14471. 10.1021/la102134w. 20715872

[ref32] Still T.; Cheng W.; Retsch M.; Jonas U.; Fytas G. Colloidal Systems: A Promising Material Class for Tailoring Sound Propagation at High Frequencies. J. Phys.: Condens. Matter 2008, 20 (40), 404203 10.1088/0953-8984/20/40/404203.

[ref33] Mock E. B.; De Bruyn H.; Hawkett B. S.; Gilbert R. G.; Zukoski C. F. Synthesis of Anisotropic Particles by Seeded Emulsion Polymerization. Langmuir 2006, 22, 4037 10.1021/la060003a. 16618142

[ref34] Mock E. B.; Zukoski C. F. Emulsion Polymerization Routes to Chemically Anisotropic Particles. Langmuir 2010, 26 (17), 13747–13750. 10.1021/la101982c. 20677747

[ref35] Chonde Y.; Krieger I. M. Emulsion Polymerization of Styrene with Ionic Comonomer in the Presence of Methanol. J. Appl. Polym. Sci. 1981, 26 (6), 1819–1827. 10.1002/app.1981.070260607.

[ref36] McMullan J. M.; Wagner N. J. Directed Self-Assembly of Colloidal Crystals by Dielectrophoretic Ordering Observed with Small Angle Neutron Scattering (SANS). Soft Matter 2010, 6 (21), 5443–5450. 10.1039/c0sm00400f.

[ref37] Panczyk M. M.; Park J. G.; Wagner N. J.; Furst E. M. Two-Dimensional Directed Assembly of Dicolloids. Langmuir 2013, 29 (1), 75–81. 10.1021/la303678f. 23215160

[ref38] Mittal M.; Furst E. M. Electric Field-Directed Convective Assembly of Ellipsoidal Colloidal Particles to Create Optically and Mechanically Anisotropic Thin Films. Adv. Funct. Mater. 2009, 19 (20), 3271–3278. 10.1002/adfm.200900908.

