# Supplementary material for: Tunable Hypersonic Bandgap Formation in Anisotropic Crystals of Dumbbell Nanoparticles
Source: ACS Nano. 2023 Sep 27;17(19):19224–31. doi: 10.1021/acsnano.3c05750 (PMC10569095; doi:10.1021/acsnano.3c05750)
Supplement: Supplementary file 1 — nn3c05750_si_001.pdf [file nn3c05750_si_001.pdf]

Supplemental Information for

Tunable hypersonic bandgap formation in anisotropic crystals of dumbbell nanoparticles

Hojin Kim[1], Abdellatif Gueddida[2], Zuyuan Wang[3], Bahram Djafari-Rouhani[2], George Fytas[3,4], and Eric M. Furst[1*]*

[1] Department of Chemical & Biomolecular Engineering, University of Delaware, Newark, Delaware 19716, USA

[2] Institut d'Electronique, de Microélectronique et de Nanotechnologie (IEMN), UMR-CNRS 8520, Département de Physique, Université de Lille, F-59655, Villeneuve d'Ascq, France

[3] Max Planck Institute for Polymer Research, Ackermannweg 10, 55128 Mainz, Germany

[4] Institute of Electronic Structure and Laser, F.O.R.T.H, 71110 Heraklion, Greece

*Corresponding Authors: fytas@mpip-mainz.mpg.de (GF) furst@udel.edu (EMF)

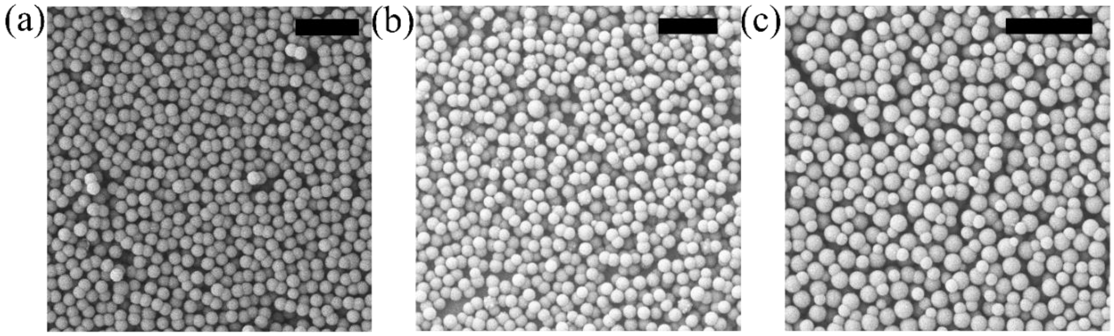

Figure S1. Scanning electron microscope images disordered colloidal films of DB1.05 (**a**), DB1.10 (b), and DB1.40 (**c**). Scale bar: 1 μm

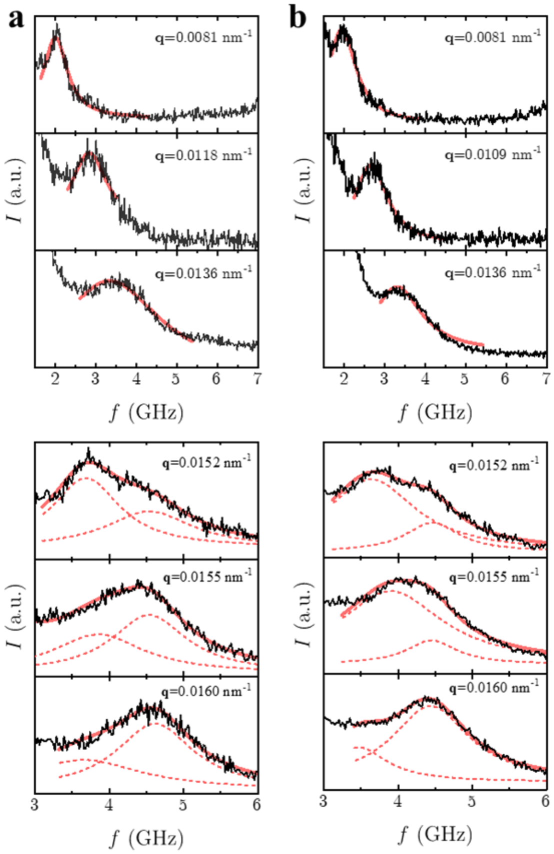

Figure S2. BLS spectra of disordered DB1.10 (**a**) and DB1.40 (**b**) particle film at various *q*. Top and bottom plots are spectra at *q* lower than q_{HG} and at *q* within the range of HG, respectively.

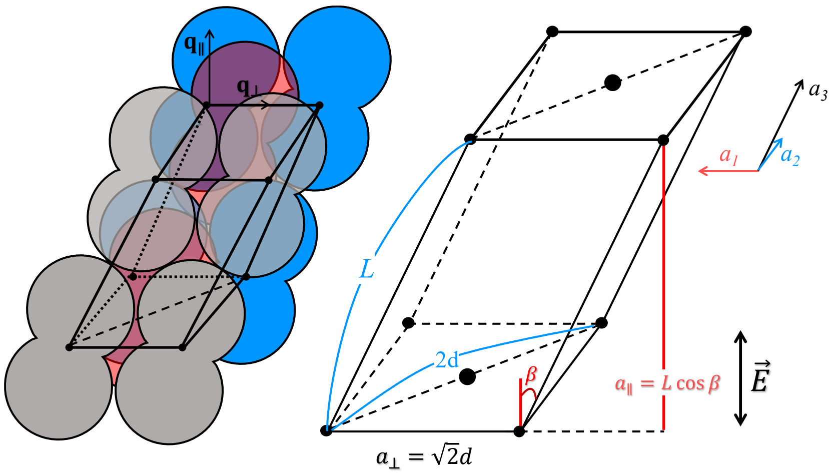

Figure S3. The schematic lattice structure of dumbbell crystal in base-centered monoclinic structure and the estimation of the lattice constant.

Table S1. The eigenfrequency of dumbbell particles in PDMS medium near the center of hybridization bandgap from FEM calculation and their vibrational mode shape indicating lateral displacements perpendicular to the particle length axis.

**Particle**	**Medium**	$f_{\mathrm{HG,\,BLS}}$ **(GHz)**	[a] $f_{2,\,\mathrm{FEM}}$ **(GHz)**	$f_{\mathrm{HG,BLS}}d/c_{\mathrm{t,PS}}$	$f_{2,\mathrm{BLS}}d/c_{\mathrm{t,PS}}$	**Mode shape**
DB1.05	PDMS	4.1	4.02	0.6683	0.6633	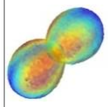
DB1.10	PDMS	4.1	3.74	0.7493	0.6919	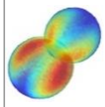
DB1.40	PDMS	4.1	4.05	0.6683	0.6683	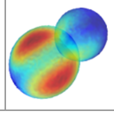

[a] The eigenfrequency of the second peak of dumbbell particles in PDMS medium.

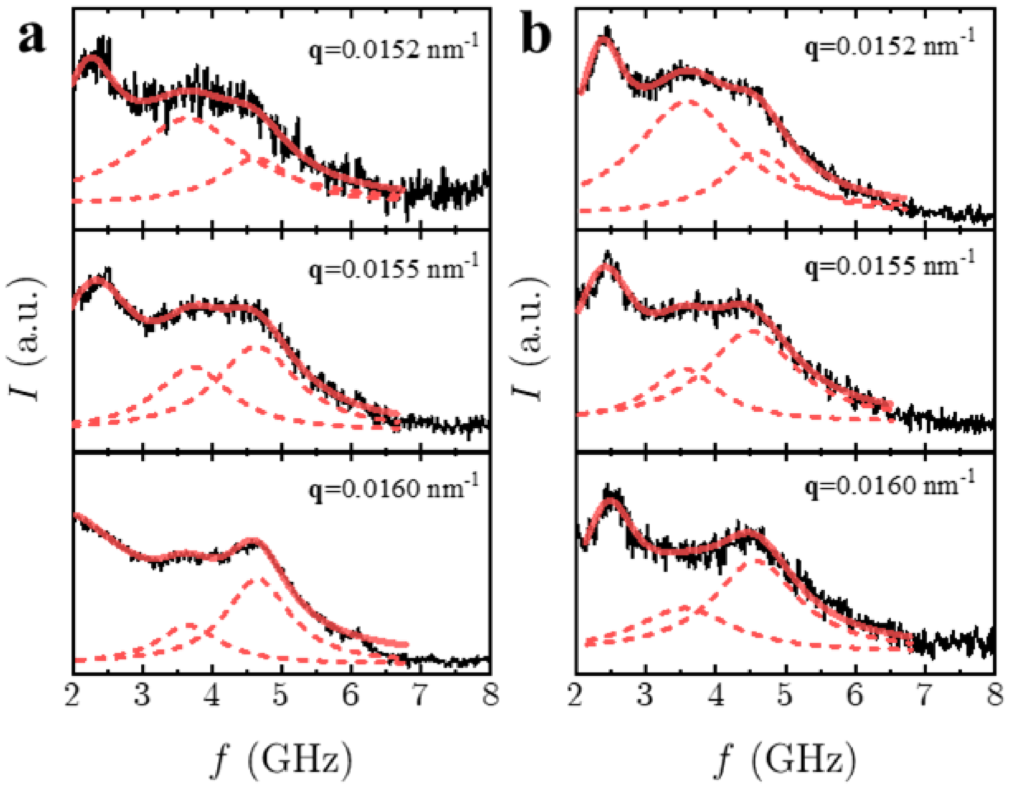

Figure S4. BLS spectra of the self-assembled colloidal crystal of dumbbells near the position of HG for **q** (**a**) parallel, $q_{\parallel}$, and (**b**) perpendicular, $q_{\perp}$, to particle length axis show peak spitting due to the formation of HG. Lower and higher peaks refer to acoustic and upper branch in the phonon band structure of the colloidal crystal, respectively.

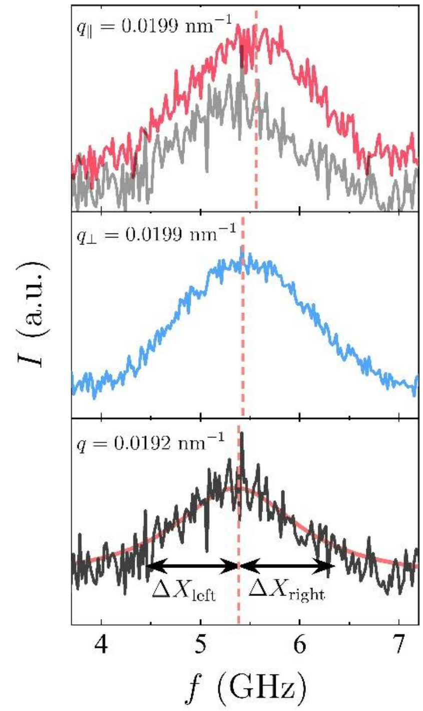

Figure S5. BLS spectra for crystal (red for $q_{\parallel}$; blue for $q_{\perp}$=0.0199 nm^{-1}) and disorder DB1.05 (bottom, q=0.0192 nm^{-1}). Despite similar q between the crystal and disorder structure, the width of the peak and symmetry exhibit significant differences due to the opening of the BG. Dashed lines refer to the center of the peak assuming a single Lorentzian peak. A single Lorentzian peak fit is shown for disordered DB1.05 (red solid line) at the bottom panel. The left (ΔX_{left}) and right ($\Delta X_{\mathrm{right}}$) peak width from the peak center indicates the skewness of peaks; (ΔX_{left}=1.22 GHz, $\Delta X_{\mathrm{right}}$=1.06 GHz for $q_{\parallel}$, ΔX_{left}=1.21 GHz, $\Delta X_{\mathrm{right}}$=1.39 GHz for $q_{\perp}$, and ΔX_{left}=0.95 GHz, $\Delta X_{\mathrm{right}}$=0.95 GHz for disordered DB1.05. The spectrum of disordered film is overlapped onto that of the crystal ($q_{\parallel}$) for the comparison.

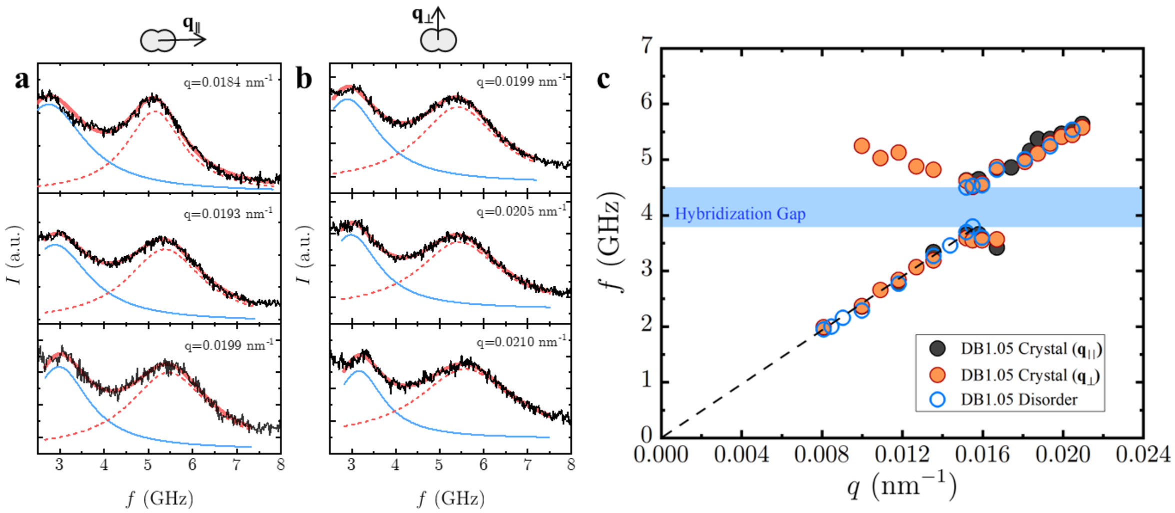

Figure S6. One-peak analysis of BLS spectra of the self-assembled DB1.05 dumbbell crystal at **q** parallel ($q_{\parallel}$) (**a**) and perpendicular ($q_{\perp}$) (**b**) to particle major axis near the Bragg bandgap. BLS spectra (black solid line) are represented (red solid line) by a single Lorentzian peak (red dashed line). The low frequency peak (blue solid line) is for the acoustic phonon of the PDMS in the crystal. Note that this single Lorentzian representation yields higher PDMS contribution as compared to the two-peak Lorentzian analysis in Figure 3a and 3b. (**c**) The phonon dispersion relation of self-assembled DB1.05 colloidal crystal recorded along ($q_{\parallel}$, black) and normal ($q_{\perp}$, orange) to the dumbbell long axis. Blue filled area indicates the hybridization bandgap along both directions. A dotted line indicates the linear q-dependence of the frequency for the acoustic branch.
